# Clinical characteristics and outcomes of diabetic ketoacidosis in patients with type 2 diabetes during acute systemic stress

**DOI:** 10.1186/s40842-026-00293-5

**Published:** 2026-06-08

**Authors:** Rania Al Sayed Abd Albaky Mohamed, Laila Mahmoud Ali Hendawy, Eman Elsayed Mahmoud Farghaly, Amr Mahmoud Mohamed Abd El Hady Saleh

**Affiliations:** Internal Medicine & Endocrine Department, Faculty of Medicine, Ain Shams University, Cairo, Egypt

**Keywords:** Diabetic ketoacidosis, COVID-19, Type 2 diabetes, Mortality, Insulin resistance, Risk factors

## Abstract

**Background:**

Many reports indicate that diabetes is one of the main risk factors for COVID-19 complications. Nevertheless, few studies have examined how DKA develops in T2DM patients who have SARS-CoV-2 infection.

**Objectives:**

This study aimed to assess β-cell function, identify risk factors for DKA, and evaluate clinical outcomes in hospitalized patients with T2DM and COVID-19.

**Methods:**

A retrospective, single-center, case–control study was conducted at Ain Shams University Isolation Hospital from August 2021 to August 2022. The study included 70 adults with T2DM and confirmed COVID-19, categorized into two groups: 35 patients with DKA (cases) and 35 patients without DKA (controls). Clinical, laboratory, and outcome data were extracted from medical records.

**Results:**

Fasting C-peptide levels did not differ significantly between the DKA and non-DKA groups (median difference: −0.06 ng/mL, 95% CI − 0.28 to 0.16; *p* = 0.363), suggesting that absolute insulin deficiency was not the primary driver of DKA in this cohort. The DKA group was significantly older (mean difference: 9.8 years, 95% CI 4.2 to 16.0; *p* = 0.003) and had a longer median diabetes duration (median difference: 3.7 years, 95% CI 1.3 to 6.1; *p* = 0.009). These patients also presented increased levels of inflammatory and stress markers, including D-dimer (mean difference: 0.21 ng/mL, 95% CI 0.05 to 0.37; *p* = 0.020) and HOMA-IR (median difference: 1.93, 95% CI 0.45 to 3.60; *p* = 0.012). Mortality was significantly greater in the DKA group (22.9% vs. 0%, risk difference 22.9%, 95% CI 8.4% to 37.4%; *p* = 0.003).

**Conclusion:**

In patients with T2DM and COVID-19, DKA was not characterized by absolute insulinopenia but was associated with older age, longer diabetes duration, severe insulin resistance, and systemic inflammation. These factors contribute to significantly increased morbidity and mortality. Our findings highlight the multifactorial nature of DKA in this setting and underscore the importance of aggressive monitoring and management in high-risk patients.

**Clinical trial number:**

Not applicable.

## Introduction

Diabetes mellitus has been associated with poor outcomes in patients with COVID-19 caused by severe acute respiratory syndrome coronavirus 2 (SARS-CoV-2**)** [1]. COVID-19 may cause new-onset diabetes or uncover diabetes that was previously undiagnosed [2]. Patients with diabetes have a greater risk of infection and acute respiratory distress syndrome than the general population does [3].

While DKA is traditionally linked to absolute insulin deficiency in type 1 diabetes(T1D), its occurrence in T2DM patients during acute illness suggests a distinct pathophysiology driven by severe insulin resistance, inflammatory stress, and relative insulin deficiency [4, 5].

Longitudinal data indicate a significant increase in the incidence of newly diagnosed T2DM during the pandemic, which is attributed to direct viral effects, systemic inflammation, glucocorticoid therapy, and pandemic-related lifestyle disruptions [6, 7]. SARS-CoV-2 may infect pancreatic β-cells via ACE2 receptors, potentially impairing insulin secretion [8, 28]. Concurrently, the cytokine storm and metabolic stress associated with severe infection can exacerbate insulin resistance and precipitate hyperglycemic crises in susceptible individuals [9]. 

Despite these associations, the specific risk factors, β-cell function, and outcomes of DKA in T2DM patients with COVID-19 remain incompletely characterized, particularly in relation to preserved β-cell function and the interplay between metabolic reserve and inflammatory stress. This study aimed to evaluate these aspects by comparing T2DM patients with COVID-19 who developed DKA to those who did not.

## Objectives 

This study aimed to assess β-cell function, identify risk factors for DKA, and evaluate clinical outcomes in hospitalized patients with T2DM and COVID-19.

### Patients and methods

#### Study design and setting

The study protocol received retrospective approval from the Institutional Review Board of Ain Shams University Faculty of Medicine (Approval No. FMASU MSO 27/2024( for analysis of anonymized clinical data collected between August 2021 and August 2022. The need for informed consent was waived per institutional policy for retrospective audit-based studies.

#### Patients

The study included 70 adult patients (≥ 18 years) with a confirmed diagnosis of T2DM and laboratory-confirmed SARS-CoV-2 infection by RT–PCR. Patients were divided into two groups:

- **Group 1 (Cases):** Thirty-five patients who developed DKA during hospitalization.
- **Group 2 (Controls):** Thirty-five patients with T2DM and COVID-19 who did not develop DKA.

DKA was diagnosed according to standard criteria: blood glucose > 200 mg/dL, arterial pH < 7.3, serum bicarbonate < 15 mEq/L, and the presence of ketonuria [10]. Ketones were measured in urine using the nitroprusside reaction test (Acetest^®^ reagent strips, Siemens Healthineers). Blood β-hydroxybutyrate measurement was not routinely available. Euglycemic DKA (blood glucose ≤ 200 mg/dL) was excluded, which may limit generalizability to contemporary presentations but ensures specificity for hyperglycemic DKA in this analysis. T2DM was diagnosed on the basis of the American Diabetes Association criteria [11]. The exclusion criteria included age < 18 years, type 1 diabetes, end-stage organ failure. Sodium-glucose cotransporter-2 inhibitor use was verified via medication reconciliation from admission records; no patients were using these medications at admission. Similarly, review of admission records confirmed that no patients were prescribed GLP-1 receptor agonists prior to or during hospitalization.

Controls (non-DKA patients) were consecutively selected from the same hospital during the study period, fulfilling the inclusion/exclusion criteria but without a diagnosis of DKA.

Notably, none of the patients in either group had a documented history of alcohol consumption or alcohol use disorder, effectively excluding alcoholic ketoacidosis as a significant confounder in this cohort.

##### Sample size:

The sample size was calculated via G*Power software (version 3.1) on the basis of a previous study reporting a 15% prevalence of DKA among hospitalized diabetic patients with COVID-19 [12]. With an α error of 0.05 and a power of 80%, a minimum of 35 patients per group was needed. This provides adequate power for primary clinical outcomes (e.g., mortality) but may be underpowered to detect subtle differences in β-cell function markers.

## Methods

### All participants underwent the following procedures

#### **History taking (either from the patients or patients’ sheets)**:

Personal history: age; sex; residency; special habits such as smoking; medical history: diabetes (duration, medications, diabetic complications); hypertension; cardiac, hepatic, and renal diseases; family history of diabetes; diabetic complications; and COVID-19 infection.

#### Clinical examination:

body weight, height, body mass index, and arterial oxygen saturation were measured via pulse oximetry.

**Chest radiology** (at baseline and when requested during follow-up): Chest X-ray, noncontrast CT.

Chest CT severity was assessed using a semi-quantitative scoring system ranging from 0 to 5. The scoring scales are based on the areal size of involvement; and the percentages and scores are designated as follows: 0 = 0% involvement; 1 = 1%–25% involvement; 2 = 26%–50% involvement; 3 = 51%–75% involvement; and 4 = 75%–100% involvement [13]. 

#### Laboratory investigations:

Blood samples for key analyses (fasting glucose, C-peptide, insulin, inflammatory markers, arterial blood gases, and electrolytes) were drawn within 24 h of admission prior to the initiation of intravenous insulin therapy for DKA patients. This timing was chosen to assess baseline metabolic status at the point of peak acute stress.

C-peptide and insulin were measured by radioimmunoassay using commercial kits (C-Peptide IRMA, DiaSorin; Insulin IRMA, Beckman Coulter). Reference ranges: C-peptide 1.1–4.4 ng/mL, insulin 2.6–24.9 µU/mL.

The homeostatic model assessment for insulin resistance (HOMA-IR) score was calculated as [fasting insulin (µU/mL) × fasting glucose (mg/dL)] / 405.

The homeostatic model assessment for beta-cell function (HOMA-B) score was calculated as [20 × fasting insulin (µU/mL)] / [fasting glucose (mg/dL) − 63].

The HOMA-IR and HOMA-B indices were originally developed for use in metabolically stable outpatient populations. Their application in acutely ill, hospitalized patients—particularly those with systemic inflammation, hypoxia, and stress hormone excess—has important limitations. Therefore, the HOMA indices in this study should be interpreted as surrogate markers rather than precise measures of insulin resistance or β-cell function.

#### Management of COVID- 19:

All patients were managed according to the national guidelines adopted by the Egyptian Ministry of Health. A national protocol for COVID-19 treatment was published first in May 2020 by the Egyptian Ministry of Health & Population, which underwent several modifications according to recent worldwide guidelines. Antibiotics were prescribed for clinical suspicion of bacterial infections as per our center’s antibiotic policy. All patients received anticoagulation (either enoxaparin or direct oral anticoagulants) unless contraindicated. Mild cases did not receive antiviral therapy, mild to moderate cases received favipiravir, and remdesivir was prescribed for those who did not respond well to favipiravir after 3 days. Patients with moderate to severe cases received remdesivir from the beginning of the study. The typical dose of favipiravir was 1600 mg/12 hours on the 1st day and 600 mg/12 hours for 4 days. The typical dose of remdesivir was 200 mg on the 1st day, followed by 100 mg/day for 4 days. Ivermectin (12 mg twice daily for 5 days) was added as a second antiviral in a few cases.

Patients with moderate-to-severe disease received steroids in the form of 80 mg/day methylprednisolone for 3–5 days. Oxygen therapy via an oxygen mask or reservoir oxygen mask, a high-flow nasal cannula, continuous positive airway pressure (CPAP) and mechanical ventilation was used according to the clinical situation and case progression [14, 15].

Quantitative data on specific pharmacologic treatments (e.g., corticosteroid dosing schedules, precise oxygen flow rates) and detailed respiratory support modalities were not systematically recorded in the clinical database available for this retrospective analysis. Consequently, these variables could not be included in the comparative analysis. This limitation is acknowledged as a critical potential source of confounding, as treatment intensity may reflect and influence both COVID-19 severity and metabolic decompensation. The study therefore focuses on characterizing the phenotype and outcomes of DKA in this setting, while explicitly acknowledging this fundamental constraint on causal inference.

#### Management of diabetic ketoacidosis (DKA):

DKA patients were managed with IV fluid, electrolyte abnormality correction, insulin therapy (IV continuous infusion) and IV sodium bicarbonate if the pH was less than or equal to 7.

#### Statistical analysis:

Data were analyzed using SPSS version 27. Continuous variables are presented as mean ± standard deviation or median (interquartile range), as appropriate, and compared using Student’s t-test or Mann–Whitney U test. Categorical variables are presented as frequencies (%) and compared using chi-square or Fisher’s exact test.

Effect sizes with 95% confidence intervals (CI) are reported for all key comparisons. To account for potential renal confounding of C-peptide, a linear regression model was constructed with C-peptide as the dependent variable and DKA status and serum creatinine as covariates. Multivariable adjustment was not performed due to the limited number of events and risk of overfitting.Missing data were minimal (< 5%) and handled by complete-case analysis.

## Results

The study included 70 patients with a mean age of 56.3 ± 13.9 years; 57.1% were female. The overall median diabetes duration was 8 years (IQR: 5–13). Hypertension was prevalent (68.6%). As shown in Table 1, the DKA group was significantly older (61.1 vs. 51.4 years, mean difference 9.8 years, 95% CI 4.2 to 16.0; *p* = 0.003) and had a longer median diabetes duration (10 vs. 7 years, median difference 3.7 years, 95% CI 1.3 to 6.1; *p* = 0.009) than the non-DKA group. No significant differences were found in sex, BMI, or baseline blood pressure. The DKA group presented with higher heart rates, respiratory rates, and body temperatures (all *p* < 0.01), indicating greater clinical severity (Table 2).

### Metabolic and laboratory parameters

Fasting C-peptide levels were comparable between the DKA and non-DKA groups (median 1.99 vs. 2.05 ng/mL), with a median difference of − 0.06 ng/mL (95% CI − 0.28 to 0.16). In contrast, insulin resistance was significantly higher in the DKA group, as reflected by higher HOMA-IR values (median difference 1.93; 95% CI 0.45–3.60). Glycemic control was poorer in the DKA group, with a mean HbA1c difference of 0.78% (95% CI 0.17–1.39) (Table 3).

After adjustment for serum creatinine, DKA status was not significantly associated with C-peptide levels (β 0.224 ng/mL; 95% CI − 0.140 to 0.587; *p* = 0.223) (Table 4).

The DKA group presented markers of greater inflammatory and thrombotic burdens, including significantly higher D-dimer levels (mean difference 0.21 ng/mL, 95% CI 0.05 to 0.37; *p* = 0.020) and more profound lymphocytopenia (median difference − 940 cells/µL, 95% CI − 1358 to − 457; *p* < 0.01).

### Outcomes

The clinical outcomes are presented in Table 5. In-hospital mortality was higher in the DKA group (22.9%) compared with the non-DKA group (0%), corresponding to an absolute risk difference of 22.9% (95% CI 8.4%–37.4%). Length of hospital stay was slightly shorter in the DKA group (mean difference − 1.4 days; 95% CI − 2.7 to − 0.1), likely reflecting early mortality.

### Risk factor analysis

Univariate logistic regression identified multiple factors associated with DKA, including age > 62 years, diabetes duration > 10 years, HbA1c > 7%, elevated HOMA-IR, and elevated D-dimer. The univariate associations should be interpreted as identifying potential risk factors rather than independent predictors (Table 6).

**Table 1:** Baseline characteristics of all studied patients (*n* = 70)

		No. = 70
**Demographics**		
Age (years)	Mean ± SD	56.26 ± 13.89
Sex	Female No. (%)	40 (57.1%)
	Male No. (%)	30 (42.9%)
Diabetic duration (years)	Median (IQR)	8 (5–13)
	Range	0.5–23
Weight (kg)	Mean ± SD	89.41 ± 10.63
BMI (kg/m2)	Mean ± SD	31.11 ± 3.39
**Associated medical disorders**		
Hypertension	No. (%)	48 (68.6%)
Cardiac diseases	No. (%)	9 (12.9%)
Acute kidney injury	No. (%)	4 (5.7%)
Chronic kidney disease	No. (%)	3 (4.3%)
Liver disease (Cirrhosis)		2 (2.8%)
Chronic obstructive pulmonary disease	No. (%)	5 (7.1%)
**Clinical data**		
Systolic BP (mmHg)	Mean ± SD	123.07 ± 17.60
Diastolic BP (mmHg)	Mean ± SD	78.21 ± 9.33
SaO2 (%)	Mean ± SD	92.29 ± 4.10

Notes: Data are presented as mean ± standard deviation (SD), median (interquartile range, IQR), or number (percentage, %) as appropriate

**Table 2:** Comparison of clinical characteristics between DKA and non-DKA groups

Variable	Group 1 (*n* = 35)	Group 2 (*n* = 35)	Effect Size (95% CI)	*p*-value
Age (years)	61.1 ± 13.5	51.4 ± 12.7	9.8 (4.2 to 16.0)	0.003
Female, n (%)	20 (57.1%)	20 (57.1%)	—	1.000
Diabetes duration (years)	10 (5–16)	7 (4–10)	3.7 (1.3 to 6.1)	0.009
Using Insulin before admission	19 (54.3%)	12 (34.3%)	—	0.09
Weight (kg)	91.3 ± 10.0	87.5 ± 11.1	3.7 (− 1.1 to 8.7)	0.142
BMI (kg/m²)	31.1 ± 3.3	31.2 ± 3.5	−0.06 (− 1.6 to 1.6)	0.937
Respiratory rate	22.8 ± 2.8	18.6 ± 5.3	4.2 (2.2 to 6.2)	< 0.001
Temperature (°C)	38.2 ± 0.5	37.6 ± 0.7	0.6 (0.3 to 0.9)	< 0.001
Heart rate (bpm)	93.3 ± 10.6	82.9 ± 9.5	10.3 (5.5 to 15.1)	< 0.001
SpO₂ (%)	92.3 ± 5.1	92.3 ± 2.8	0.1 (− 1.5 to 1.6)	0.954
CT chest score	4.57 ± 0.56	4.74 ± 0.51	—	0.184

Notes: Continuous variables are expressed as mean ± SD or median (IQR). Between-group comparisons were performed using Student’s t-test for normally distributed variables and Mann–Whitney U test for non-normally distributed variables. Categorical variables were compared using chi-square or Fisher’s exact test

*Hodges–Lehmann median difference and 95% confidence interval are reported for non-normally distributed variables.CI = confidence interval

**Table 3:** Comparison of laboratory parameters between DKA and non-DKA groups

Parameter	Group 1 (*n* = 35)	Group 2 (*n* = 35)	Effect Size (95% CI)	*p*-value
Hemoglobin (g/dL)	12.24 ± 1.74	13.00 ± 1.09	−0.76 (− 1.43 to − 0.11)	0.031
Leucocyte (×10⁹/L)	10.0 (7.1–15.7)	13.1 (9.3–15.6)	−3.1 (− 5.9 to − 0.1)	0.042
Lymphocytes (×10⁹/L)	550 (258–900)	1490 (900–2150)	−940 (− 1358 to − 457)	< 0.001
FPG(mg/dL)	170.5 ± 27.6	138.7 ± 23.3	31.8 (20.2 to 44.9)	< 0.001
HbA1c (%)	7.22 ± 1.74	6.44 ± 0.50	0.78 (0.17 to 1.39)	0.013
Fasting C-peptide (ng/mL)	1.99 (1.56–2.45)	2.05 (1.70–2.53)	−0.06 (− 0.28 to 0.16)	0.363
HOMA-IR	5.33 (3.66–8.75)	3.40 (2.10–6.90)	1.93 (0.45 to 3.60)	0.012
HOMA-B	30.49 (21.69–43.35)	57.24 (33.16–89.06)	-25.2(-42.22 to – 8.5)	0.004
D-dimer (ng/mL)	0.87 ± 0.44	0.66 ± 0.26	0.21 (0.05 to 0.37)	0.020
pH	7.31 ± 0.07	7.39 ± 0.03	−0.08 (− 0.11 to − 0.06)	< 0.001
HCO₃ (mEq/L)	17.40 ± 3.31	23.68 ± 1.29	−6.28 (− 7.47 to − 5.10)	< 0.001
Lactate (mmol/L)	2.5(IQR:1.3–2.9)	2.2 (IQR:1.7–2.4)	0.2 ( -0.34 to 0.74)	0.888
Potassium (mmol/L)	3.61 ± 0.95	4.32 ± 0.66	−0.71 (− 1.11 to − 0.35)	< 0.001

Notes: Data are presented as mean ± SD or median (IQR). Comparisons were made using Student’s t-test or Mann–Whitney U test as appropriate. Effect sizes are expressed as mean differences or Hodges–Lehmann median differences* with 95% CIs

FPG = fasting plasma glucose; HOMA-IR = homeostatic model assessment of insulin resistance

**Table 4:** Creatinine-adjusted analysis of fasting C-peptide

Predictor	B (unstandardized)	95% Confidence Interval	*p*-value
DKA (yes vs. no)	0.224	−0.140 to 0.587	0.223
Serum creatinine (mg/dL)	0.290	−0.067 to 0.647	0.109

Notes: Linear regression model with fasting C-peptide as the dependent variable. Model statistics: R² = 0.055, Adjusted R² = 0.023, ANOVA *p* = 0.185

**Table 5:** Clinical outcomes between DKA and Non-DKA groups

Outcome	Group 1 (*n* = 35)	Group 2 (*n* = 35)	Effect Size (95% CI)	*p*-value
In-hospital mortality, n (%)	8 (22.9%)	0 (0%)	22.9% (8.4% to 37.4%)	0.003
Length of stay (days)	8.1 ± 3.1	9.5 ± 2.4	−1.4 (− 2.7 to − 0.1)	0.037

Notes: Mortality compared using Fisher’s exact test; length of stay compared using Student’s t-test. Effect sizes are risk difference (%) and mean difference with 95% CI

**Table 6:** Univariate logistic regression for factors associated with DKA

Factor	OR (95% CI)	*p*-value
Age > 62 years	5.74 (1.91–17.28)	0.002
Diabetes duration > 10 years	20.09 (2.45–164.64)	0.005
HbA1c > 7%	15.58 (3.23–75.18)	0.001
HOMA-IR > 3.5	5.74 (1.91–17.28)	0.002
D-dimer > 0.77 ng/mL	3.43 (1.25–9.40)	0.017

Notes: OR = odds ratio; CI = confidence interval. Univariate logistic regression was performed for each variable

## Discussion

This retrospective case–control study provides critical insights into the pathogenesis, risk factors, and clinical outcomes of diabetic ketoacidosis (DKA) in patients with type 2 diabetes mellitus (T2DM) during acute SARS-CoV-2 infection. Our findings challenge the conventional paradigm of absolute insulin deficiency as the primary driver of DKA in this population and instead highlight a multifactorial pathophysiology involving severe insulin resistance, preexisting metabolic vulnerability, and systemic inflammatory stress.

### Preserved β-cell function and pathophysiological implications

The most significant finding of our study was the comparable fasting C-peptide levels between the DKA and non-DKA groups (median 1.99 vs. 2.05 ng/mL, *p* = 0.363), suggesting that absolute insulin deficiency—characteristic of type 1 diabetes-related DKA—was not the predominant mechanism in T2DM patients with COVID-19. This observation remained non-significant after adjustment for serum creatinine (β 0.224, *p* = 0.223), suggesting that group differences in renal function did not account for the lack of difference in C-peptide levels between groups. This aligns with the emerging concept of “ketosis-prone type 2 diabetes” (KPD), where patients with phenotypic T2DM develop DKA under conditions of extreme metabolic stress despite having preserved endogenous insulin secretory capacity [16]. The pathophysiology appears to involve a dual insult: severe insulin resistance induced by the cytokine storm and counterregulatory hormone excess of COVID-19, coupled with relative insulin deficiency in the context of long-standing β-cell exhaustion [17]. Our finding of significantly higher HOMA-IR in the DKA group (5.33 vs. 3.40, *p* = 0.012) supports this hypothesis, although we emphasize the limitations of HOMA-IR as a surrogate marker in this acutely ill cohort [18].

The preserved C-peptide levels must be interpreted cautiously. While they suggest adequate β-cell mass, they do not exclude functional β-cell impairment under acute stress. SARS-CoV-2 has been shown to infect pancreatic β-cells through ACE2 receptors, potentially causing transient dysfunction [19]. Additionally, the timing of measurement is critical; our samples were drawn at admission before significant insulin therapy, but the dynamic nature of insulin secretion during evolving DKA could not be captured. Nonetheless, our data contribute to the growing evidence that DKA in T2DM patients during the COVID-19 pandemic represents a distinct entity from classic T1D DKA, with important therapeutic implications.

### Risk factor profile: metabolic vulnerability meets inflammatory storm

Univariate analysis showed that longer diabetes duration (> 10 years) was strongly associated with the development of DKA (OR: 20.09, 95% CI: 2.45–164.64, *p* = 0.005). This finding underscores the concept of “metabolic reserve” exhaustion, where patients with long-standing T2DM have diminished β-cell functional capacity to compensate for acute insulin resistance [20]. The DKA group also exhibited poorer chronic glycemic control (HbA1c 7.22% vs. 6.44%, *p* = 0.013), which may reflect both disease severity and therapeutic inertia prior to hospitalization.

The DKA group demonstrated a pronounced proinflammatory and prothrombotic state, with significantly elevated D-dimer (0.87 vs. 0.66 ng/mL, *p* = 0.020) and profound lymphocytopenia (median 550 vs. 1490 cells/µL, *p* < 0.01). These markers likely reflect greater overall COVID-19 severity than direct causative factors for DKA. The bidirectional relationship between hyperglycemia and inflammation is well established: hyperglycemia promotes a proinflammatory state through mitochondrial reactive oxygen species generation and advanced glycation end-product formation, whereas cytokines such as TNF-α and IL-6 directly induce insulin resistance [21]. This creates a vicious cycle in which COVID-19-induced inflammation worsens hyperglycemia, which in turn amplifies the inflammatory response.

Notably, emerging evidence suggests that anti-inflammatory interventions may mitigate metabolic dysregulation in patients with COVID-19. Observational studies indicate that aspirin use is associated with a reduced incidence of new-onset diabetes following SARS-CoV-2 infection, potentially through the modulation of NLRP3 inflammasome activity and platelet-mediated inflammation [22, 23, 39]. While our study design did not allow assessment of the effects of aspirin, this represents a promising avenue for future intervention studies in high-risk populations.

### Clinical outcomes and mortality determinants

The notably higher mortality in the DKA group (22.9% vs. 0%, *p* = 0.003) highlights the lethal synergy between metabolic decompensation and viral illness. Importantly, this excess mortality cannot be attributed solely to more severe COVID-19 pneumonia, as CT chest severity scores were comparable between groups (4.57 ± 0.56 vs. 4.74 ± 0.51, respectively; *p* = 0.182). This finding indicates that the degree of pulmonary involvement at admission was similar in both groups. Therefore, the increased mortality in DKA patients likely reflects the compounded burden of metabolic acidosis, severe insulin resistance, and the proinflammatory state characteristic of DKA superimposed on the viral infection, rather than more extensive lung damage alone. This mortality rate is consistent with previous reports; Stevens et al. [24] reported 36.9% mortality in COVID-19 patients with DKA versus 28.8% without DKA, whereas Pal et al. reported mortality rates approaching 50% in systematic reviews [25].

The paradoxically shorter hospital stay in the DKA group (8.1 vs. 9.5 days, *p* = 0.037) likely reflects higher early mortality rather than more rapid recovery, a phenomenon observed in other studies of critical illness [26]. This finding underscores the need for aggressive early intervention in DKA patients with COVID-19, as the window for effective treatment may be narrow.

Our study contributes to the growing body of evidence that SARS-CoV-2 infection serves as a potent metabolic stress test, unmasking and exacerbating underlying dysglycemia. Longitudinal cohort studies have documented a significant increase in the incidence of new-onset type 2 diabetes during the pandemic [27, 40]. Multiple mechanisms likely contribute directly to viral damage to β-cells [19], inflammation-induced insulin resistance [21], glucocorticoid therapy [29], and pandemic-related lifestyle disruptions [30]. Our findings, specifically in patients with preexisting T2DM, suggest that those with longer disease duration and poorer control are particularly vulnerable to this decompensation.

### COVID-19 as a catalyst for metabolic decompensation

The intersection of COVID-19, diabetes, and DKA exists within the broader context of CKM syndrome, which recognizes the interconnected pathophysiology of cardiovascular, kidney, and metabolic diseases [31]. Emerging evidence suggests that SARS-CoV-2 infection may accelerate CKM progression through multiple pathways: endothelial dysfunction, persistent low-grade inflammation, and immune dysregulation [32]. Furthermore, molecular analyses of long COVID suggest persistent viral reservoirs, autoimmunity, and chronic inflammation may contribute to new-onset diabetes, highlighting long-term pathobiological relationships [38]. Patients who survive DKA during COVID-19 may represent a high-risk subgroup requiring intensified long-term CKM monitoring and management. Future studies should investigate whether acute metabolic decompensation during COVID-19 predicts accelerated progression of microvascular and macrovascular complications. This concern is magnified by data indicating the COVID-19 pandemic has significantly increased the global burden of type 2 diabetes [41].

### Comparison with existing literature

Our demographic findings align with those of several previous studies. The older age and longer diabetes duration in our DKA group mirror observations by Goldman et al. [12] and Dell’Aquila et al. [32], who reported similar risk profiles. The absence of sex predominance in our study contrasts with some Western cohorts showing male predominance [24, 33], possibly reflecting regional differences in diabetes epidemiology and healthcare access.

Our laboratory findings corroborate previous reports of elevated inflammatory markers in DKA patients with COVID-19. Similarly, Mondal et al. [34] reported increased D-dimer levels in DKA patients, whereas Meza et al. [35] reported profound lymphocytopenia in a case series. The electrolyte abnormalities we observed, particularly hypokalemia, are consistent with Reddy et al.‘s early case reports [36] and Kempegowda et al.‘s comparative study [37].

### Strengths and limitations

The strengths of our study include its case–control design with carefully defined criteria, comprehensive laboratory assessment including β-cell function markers, and real-world management reflecting contemporary COVID-19 care. To our knowledge, this is the first study to systematically evaluate fasting insulin and C-peptide levels in patients with T2DM, COVID-19, and DKA, providing direct insights into the pathophysiological mechanisms underlying metabolic decompensation in this population. The inclusion of a control group of T2DM patients with COVID-19 but without DKA provides valuable comparative data lacking in many case series. Our findings challenge the traditional paradigm of absolute insulin deficiency in DKA and contribute to the evolving understanding of ketosis-prone diabetes phenotypes.

However, several limitations must be acknowledged. First, theretrospective, single-center design introduces potential selection bias and limits generalizability. Second, the relatively small sample size, while adequate for primary outcomes, reduces statistical power for secondary and multivariable analyses and inflates the uncertainty around some effect estimates. Third, significant baseline differences existed between groups (age, diabetes duration); however, only descriptive and univariate analyses were performed, and residual confounding remains likely. Fourth, and most critically, the absence of detailed data on COVID-19-specific treatments (corticosteroid dosing, antiviral agents, intensity of respiratory support) represents a major unmeasured confounder. The DKA group presented with greater clinical severity, making it highly probable they received more intensive immunomodulatory and respiratory support. Since corticosteroids are potent inducters of insulin resistance and hyperglycemia, this confounding severely limits our ability to disentangle the independent contributions of DKA, severe COVID-19, and its treatment to the observed outcomes. This limitation fundamentally constrains causal inference; our results should be interpreted as characterizing a high-risk clinical phenotype rather than establishing independent causal pathways. Fifth, the cross-sectional assessment of β-cell function provides only a snapshot; longitudinal measurements would better characterize dynamic changes. Sixth, the diagnosis of DKA relied on urinary ketone testing via the nitroprusside reaction, which semi-quantitatively measures acetoacetate but not the predominant ketone body, β-hydroxybutyrate. The absence of systematic serum β-hydroxybutyrate measurement may affect the precision of diagnosis, severity assessment, and monitoring of resolution. This is a recognized limitation of retrospective studies conducted in resource-constrained settings during the peak of the pandemic. Seventh, the exclusion of euglycemic DKA, While we systematically excluded patients using SGLT2 inhibitors and confirmed no use of GLP-1 receptor agonists in our cohort, clinicians should remain aware that GLP-1 RAs, particularly during intercurrent illness with poor oral intake or dehydration, have been rarely implicated in ketoacidosis. Future studies with larger sample sizes should consider stratifying by all anti-diabetic medication classes. Finally, the lack of longitudinal follow-up precludes assessment of post-discharge metabolic outcomes and long-term cardiovascular or renal sequelae in this high-risk population.

### Clinical implications and future directions

Our findings have several practical implications. First, they underscore the need for vigilant glucose monitoring in hospitalized T2DM patients with COVID-19, particularly those with longer disease durations and suboptimal control. Second, they suggest that management strategies should address both hyperglycemia and the underlying inflammatory state. Third, they highlight the importance of multidisciplinary care that integrates endocrinology, infectious disease, and critical care expertise.

Future research should focus on several areas: prospective validation of our risk factors in larger multicenter cohorts; investigation of optimal insulin regimens for COVID-19-related DKA; assessment of anti-inflammatory agents (including aspirin) for metabolic protection; and longitudinal studies of CKM outcomes in survivors. Additionally, mechanistic studies exploring the interplay among SARS-CoV-2, insulin signaling pathways, and ketogenesis are needed to elucidate the pathophysiology fully.

## Conclusion

In conclusion, our study demonstrated that DKA in patients with T2DM during COVID-19 represents a distinct clinical entity characterized by preserved insulin secretion but severe insulin resistance in the context of systemic inflammation. Longer diabetes duration emerged as the most consistently associated clinical characteristic among patients who developed DKA, highlighting the importance of chronic disease management in pandemic preparedness. The high mortality observed underscore the critical need for early recognition and aggressive management of this dual metabolic–infectious emergency. As we continue to navigate the long-term consequences of the pandemic, understanding these complex interactions will be essential for optimizing care for patients with diabetes facing acute systemic stressors.

## Data Availability

Data are available on request.

## Code availability

The datasets generated and/or analyzed during the current study are available from the corresponding author upon reasonable request. This study did not generate or utilize custom code or software algorithms. Standard statistical software (SPSS version 27) was used for data analysis as described in the Methods section.
